# Identification of Candidate Serum Proteins for Classifying Well-Differentiated Small Intestinal Neuroendocrine Tumors

**DOI:** 10.1371/journal.pone.0081712

**Published:** 2013-11-25

**Authors:** Spyros Darmanis, Tao Cui, Kimi Drobin, Su-Chen Li, Kjell Öberg, Peter Nilsson, Jochen M. Schwenk, Valeria Giandomenico

**Affiliations:** 1 Department of Immunology, Genetics, and Pathology, Uppsala University, Uppsala, Sweden; 2 Department of Medical Sciences, Endocrine Oncology, Uppsala University, Uppsala, Sweden; 3 Clinic of Endocrine Oncology, Uppsala University Hospital, Uppsala, Sweden; 4 Science for Life Laboratory, School of Biotechnology, KTH - Royal Institute of Technology, Stockholm, Sweden; 5 Science for Life Laboratory, Uppsala, Sweden; Baylor College of Medicine, United States of America

## Abstract

**Background:**

Patients with well-differentiated small intestine neuroendocrine tumors (WD-SI-NETs) are most often diagnosed at a metastatic stage of disease, which reduces possibilities for a curative treatment. Thus new approaches for earlier detection and improved monitoring of the disease are required.

**Materials and Methods:**

Suspension bead arrays targeting 124 unique proteins with antibodies from the Human Protein Atlas were used to profile biotinylated serum samples. Discoveries from a cohort of 77 individuals were followed up in a cohort of 132 individuals both including healthy controls as well as patients with untreated primary WD-SI-NETs, lymph node metastases and liver metastases.

**Results:**

A set of 20 antibodies suggested promising proteins for further verification based on technically verified statistical significance. Proceeding, we assessed the classification performance in an independent cohort of patient serum, achieving, classification accuracy of up to 85% with different subsets of antibodies in respective pairwise group comparisons. The protein profiles of nine targets, namely IGFBP2, IGF1, SHKBP1, ETS1, IL1α, STX2, MAML3, EGR3 and XIAP were verified as significant contributors to tumor classification.

**Conclusions:**

We propose new potential protein biomarker candidates for classifying WD-SI-NETs at different stage of disease. Further evaluation of these proteins in larger sample sets and with alternative approaches is needed in order to further improve our understanding of their functional relation to WD-SI-NETs and their eventual use in diagnostics.

## Introduction

Neuroendocrine tumors (NETs) are rare, life-threatening, malignant solid tumors, which arise in hormone-secreting tissue of the diffuse neuroendocrine system. During the early stages of disease, NETs are generally slow-growing and asymptomatic, whereas at a later stage, tumor metastasis to the liver appears along with hormonal hypersecretion. This generally leads to well defined and debilitating clinical syndromes such as the flushing and diarrhea of the carcinoid syndrome. Although several guidelines have been agreed on to standardize diagnosis, due to the insidious natural history of NETs, diagnosis is still made after tumors produce clinical symptoms and are metastatic [1]. In particular, well-differentiated small intestinal neuroendocrine tumor (WD-SI-NET) patients are predominantly diagnosed with a delay of three to four years at a metastatic stage of the disease, hindering possible curative treatment.

Several variables, such as the rarity and heterogeneity of these malignancies, the multiplicity of NET classification systems and the historical lack of well-designed clinical trials may contribute to the diagnostic delay. It has been previously suggested that a better understanding of NET biology, blood biomarkers, and improved analytical approaches to identify tumors, localizations and small lesions [2] are required to achieve improved outcomes in NETs.

The goal of the presented study was to discover candidate biomarker protein profiles for WD-SI-NETs, by investigating proteomic signatures in serum of WD-SI-NET patients and healthy individuals. We used a highly multiplexed antibody suspension bead array [3]-[5] targeting 124 unique proteins with 184 antibodies produced and validated in the context of the Human Protein Atlas (HPA) [6] in an initial sample cohort of 20 healthy individuals and 57 WD-SI-NET patients at different stages of disease. We were able to identify 20 interesting putative biomarkers that were further validated in a second cohort of 36 healthy individuals and 96 WD-SI-NET patients. Moreover, we discovered sets of protein profiles that discriminate healthy individuals from WD-SI-NET patients at different stages of disease with a classification accuracy of up to 85%.

## Results

**Figure 1:**
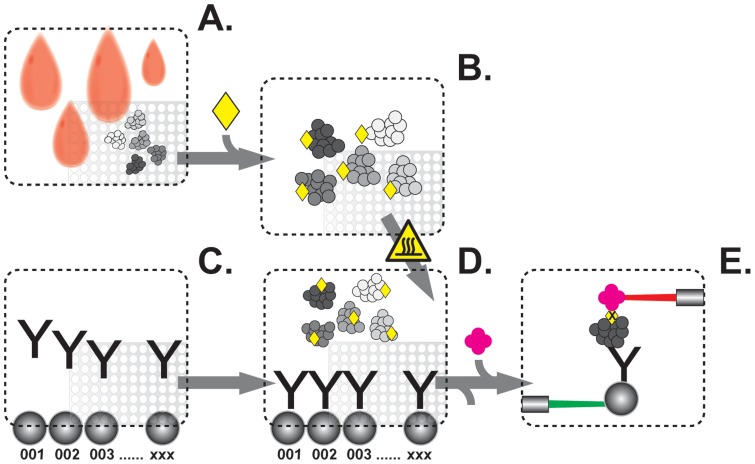
Experimental procedure of antibody suspension bead arrays. The process starts with the distribution of samples into microtiter plates according to a defined, randomized sample positioning (A). The protein content of diluted samples is then labeled with biotin (B) and antibodies are coupled onto beads with distinct color codes to create a suspension bead array (C). Beads and samples are combined for incubation after the samples have been heat treated in assay buffer (D). Proteins that have not been captured by antibodies are removed and fluorescent streptavidin is added for detection (D). The beads are then measured and the co-occurrence of beads, which are identified via a green laser, and the emitted reporter fluorescence, excited by a red laser, allow the determination of interaction dependent intensity values in multiplex (E).

This study aims at expanding the list of potential biomarkers for classifying WD-SI-NETs at different stages of disease using proteomic signatures generated in serum samples by highly multiplexed antibody suspension bead arrays (**Figure 1**). We divided the serum samples into two independent sample sets, further called cohort 1 and 2, consisting of 77 and 132 samples respectively. We used cohort 1 to screen 124 protein candidates and selected a subset of those for further analysis based on their significance using a Wilcoxon rank sum test and their importance as classifiers using multivariate classification. Analytes selected in cohort 1 were then followed up in a subsequent verification in cohort 2.

### Discovery of candidate protein profiles

**Figure 2:**
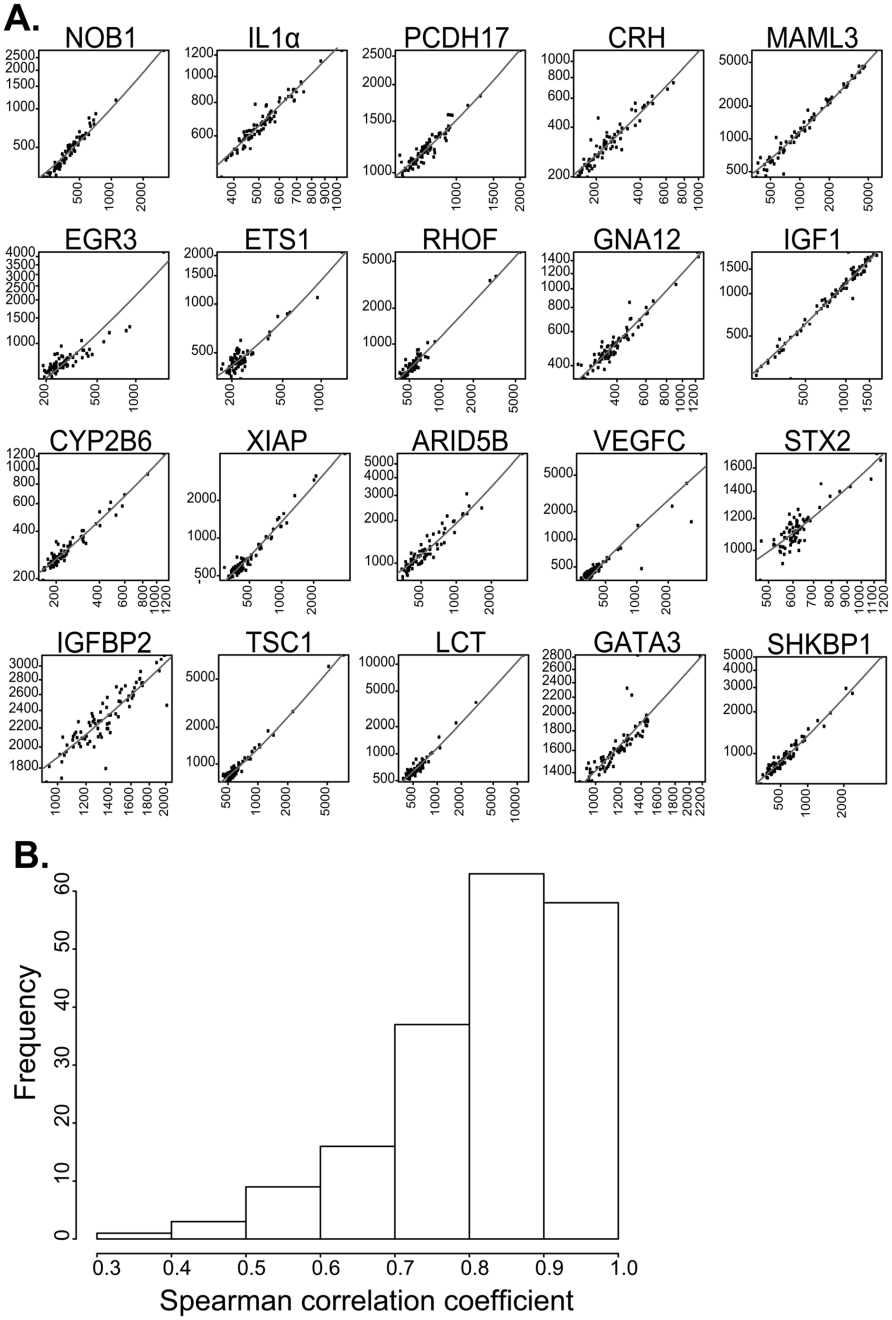
Correlation analysis during the discovery phase. Correlation of two independent experiments for all 20 selected analytes (A). Each data point represents 1 of 77 patients and controls included in cohort 1. Axes represent MFI for each sample. Correlation analysis between two independent experiments of all the 124 analytes (B). X-axis represents Spearman correlation coefficients, whereas the y-axis represents frequency.

We assessed the profile levels of 124 proteins in a sample cohort comprising 57 WD-SI-NET patients at different stages and 20 healthy controls (cohort 1). A detailed overview of cohort 1 can be found in **Table 1**. All samples were analyzed in multiple independent measurements to assess reproducibility of the single binder assay. The raw data on cohort 1 samples, obtained by two measurements, were deposited in **Table S1a** and S**1b**, respectively. We found that the assays exhibited high inter-experimental Spearman correlation coefficients across samples of rho >0.9 as shown in **Figure 2b** **.**

**Table 1:** Descriptive statistics for each of the two cohorts used in the study.

Cohort 1	N	Age (mean±SE)	Gender		Cohort 2	N	Age (mean±SE)	Gender	
			M	F				M	F
HC	20	54±2	10	10	HC	36	56±2	18	18
PT	19	56±3	15	4	PT	29	69±2	18	11
LNM	19	65±3	8	11	LNM	23	63±2	14	9
LM	19	64±3	7	12	LM	44	64±2	15	29

To select candidates for further analysis, we used a Wilcoxon rank sum test and selected analytes with p values smaller than 0.01. In addition, we performed multivariate random forest (RF) [7] and between group analysis (BGA) [8] for the classification of different groups identifying the most important analytes for each classification. Since no multiple-testing correction was used during the screening phase, identified candidates were required to arise as either significant or important, for the univariate and multivariate analysis respectively, in every independent experimental analysis (technical verification). A first list of protein profiles from 20 antibodies was generated during the discovery phase, summarized in **Table S2.** To describe the inter-assay concordance, correlations between two independent measurements for each of the 20 selected analytes are presented in **Figure 2a**.

### Verification of global analysis

The selected 20 analytes were further investigated in a larger sample cohort of 36 healthy individuals and 96 cancer patients (cohort 2) described in **Table 1**. The raw data on cohort 2 samples were deposited in **Table S1c**.

**Figure 3:**
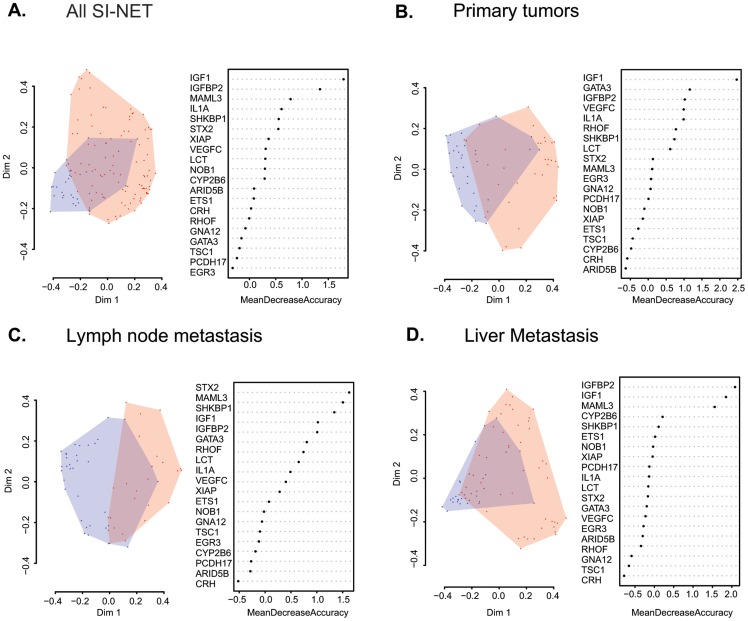
Multivariate classification of different individual groups. Distribution of each individual sample belonging to the healthy control group (blue circles) and all SI-NET patients (A), patients with primary tumors (B), lymph node metastasis (C), and liver metastasis (D). Axes values correspond to the two-dimensional projection of the proximity matrices generated for each pairwise comparison using RF. For each group, the relative importance of each protein used in the multivariate classification is shown.

We performed multivariate classification using random forest (RF) analysis in two different ways: First, we used RF analysis to estimate proximity between different samples based on the abundance of each of the 20 previously selected markers. Subsequently, we calculated the scaling coordinates of the RF-derived proximity matrix thus reducing the dimensions by which each sample is represented to two. A typical two-dimensional representation of different samples is shown in **Figure 3**, along with a measure of the relative importance of each of the proteins for the classification. We then calculated the classification performance by assigning a class to each sample based on its proximity with samples of a similar class (5-nearest neighbor classification). Results showed that using the selected set of 20 antibodie classes were assigned correctly in 86% of the cases (sensitivity = 92%, specificity = 72%).

**Figure 4:**
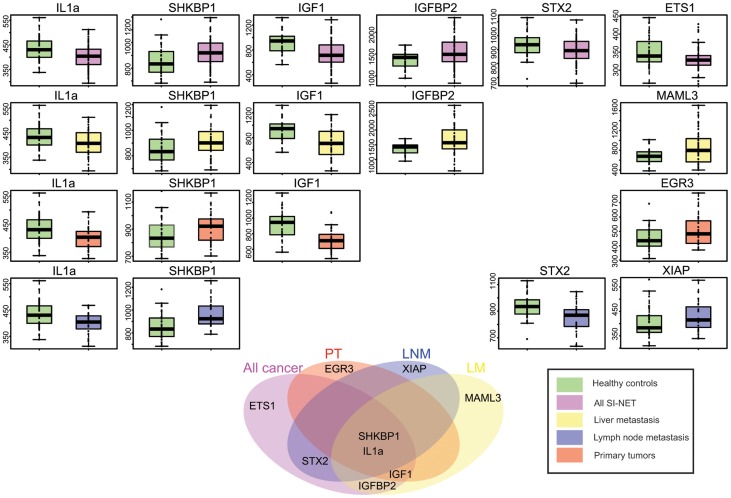
Group comparison by using the 9 selected proteins. Boxplots and ROC curves for each of the significant analytes in each of the pairwise comparisons between Healthy individuals (green color) and all cancer patients combined (All SI-NET), Liver metastasis (LM), Primary tumors (PT) and Lymph-node metastasis (LNM). In the lower part of Figure 3, a Venn diagram for all significant analytes is presented. Common analytes for the discrimination of each cancer patient group and healthy controls are shown.

Having performed multivariate classification using all 20 antibodies, we investigated the association of each of the 20 analytes with disease state by a univariate Wilcoxon test. Antibodies towards six proteins, namely insulin-like growth factor-binding protein 2 (IGFBP2), SH3KBP1-binding protein 1 (SHKBP1), protein C-ets-1 (ETS1), insulin-like growth factor I (IGF1), interleukin 1 alpha (IL1α), and syntaxin-2 (STX2), appeared as significant (p<0.01) when comparing all SI-NET samples at different stages of disease with healthy controls. In **Figure 4A** **,** profiles from each of these antibodies are shown. For every protein, AUC values were calculated and are presented in **Table 2**.

**Table 2:** AUC values for every significant analyte.

PT		LNM		LM		All SI-NET	
Protein	AUC	Protein	AUC	Protein	AUC	Protein	AUC
IGF1	0.81	SHKBP1	0.74	IGF1	0.74	IGF1	0.74
IL1α	0.69	STX2	0.73	IGFBP2	0.73	SHKBP1	0.67
SHKBP1	0.65	IL1α	0.69	SHKBP1	0.64	IL1α	0.67
EGR3	0.63	XIAP	0.63	IL1α	0.64	IGFBP2	0.66
				MAML3	0.64	ETS1	0.62
						STX2	0.61

In addition, the combined AUC value of all proteins for every group is presented.

### Verification of tumor type specific analysis

We performed multivariate classification on healthy individuals and patients with primary tumors from cohort 2, using all 20 selected antibodies as described above. Thus, correct classification to each sample was achieved with an 85% success rate (sensitivity = 83%, specificity = 86%). When comparing healthy individuals to patients with primary tumors, four out of 20 protein profiles were found significant (p<0.01) in cohort 2, namely IGF1, IL1α, SHKBP1, and early growth response protein 3 (EGR3).

We performed an additional analysis, comparing healthy individuals to patients with liver metastasis (LM) and lymph node metastasis (LNM). For 87% of the samples belonging to the LM group a correct class assignment was calculated using all 20 previously protein profiles (sensitivity = 85%, specificity = 86%). Furthermore, we identified individual proteins IGF1, IGFBP2, IL1α, mastermind-like protein 3 (MAML3), and SHKBP1 as significant (p<0.01) in cohort 2.

For LNM patients, we determined a classification correctness of 84% (sensitivity = 83%, specificity = 80%) with samples of cohort 2. For the same patient group, we identified 4 protein profiles as significant (p<0.01) in cohort 2, namely for the targets IL1α, SHKBP1, STX2, and X-linked inhibitor of apoptosis (XIAP). In **Figure 4E**, an overview of significant profiles is shown for all the pairwise comparisons. Protein profiles for targets such as IL1α and SHKBP1 were significantly different between healthy individuals and each of the primary tumor (PT), lymph node metastasis (LNM), and liver metastasis (LM) patients whereas protein profiles for MAML3 were unique for the classification of LM patients, XIAP for the classification of LNM patients, and EGR3 for PT patients.

**Figure 5:**
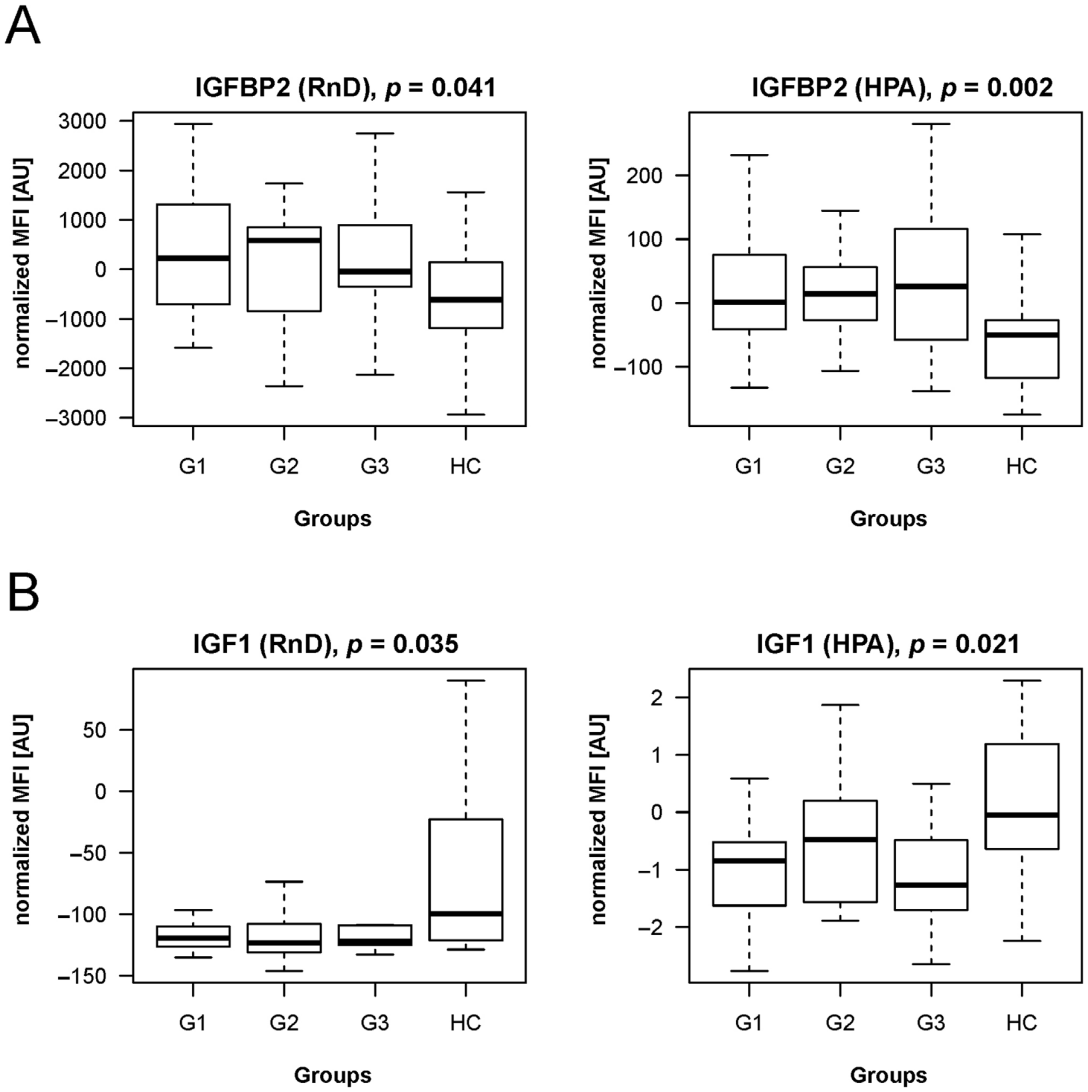
IGFBP2 and IGF1 sandwich immunoassays. Commercially available sandwich immunoassays were performed to confirm the differential detection of IGFBP2 (A) and IGF1 (B). The assays were supplemented with HPA045140 for IGFBP2 and with HPA048946 for IGF1, both used during discovery. In the two assays both capture antibodies revealed concordant and previously observed differences between cases and control group (p<0.05). Data shown was normalized using a linear model.

To confirm some of the findings, we proceeded by analyzing a subset of patients and controls from cohort 2 (n = 95) using sandwich immunoassays for IGF1 and IGFBP2. Assays were performed by using previously employed HPA antibodies in parallel with capture antibody of the sandwich pair. In these analyses, both capture antibodies revealed a concordant and significant (p<0.05) difference in abundance of IGF1 (decreased in cancer) and IGFBP2 (increased in cancer), as shown in **(** **Figure 5A and B** **)**. Profiles from antibodies used for IGFBP2 correlated well (rho = 0.7), whereas by using HPA048946 antibody targeting IGF1, the signals above background were exclusively detected in the healthy control samples, thus compromising this correlation (rho = 0.3).

## Discussion

WD-SI-NETs produce and secret various amines and peptides, which can be used as markers locally in tissue [9] or in body fluids such as blood [10]. Chromogranin A (CgA) is the most commonly used general tumor marker at the moment. CgA is expressed in 80-90% of all patients with gastrointestinal pancreatic-NETs, which comprise WD-SI-NETs. Although CgA works well for the diagnosis of NETs, it is not a relevant biomarker at the stage of metastatic disease, a stage for which we miss curative therapies. The unmet need of recognition and identification of primary SI-NETs requires further investigation to identify novel specific biomarkers for the identification of tumors in the early phase of malignancy. Along these lines, we recently identified autoantibodies against the paraneoplastic MA antigen 2, which may be important to detect patient recurrences [11], as well as olfactory receptor 51E1 as a new potential tissue biomarker for these tumors [12] and SI-NETs differentially expressed microRNAs [13].

The presented study is an exploratory approach using antibody suspension bead arrays on a collection of serum samples from WD-SI-NET patients at different stages of disease. All antibodies used were routinely validated for specificity using planar protein microarrays against 384 protein antigens [14] as well as other methods [15]. The analytical format used here is a highly multiplexed single-binder immunoassay to enrich a protein in a complex solution, which yet cannot exclude off-target binding events. Such events stem from weak affinity interactions to more abundant target proteins than the ones addressed by the used antibody. Our strategy here was to increase confidence in on-target binding through (i) re-analysis, (ii) analysis of additional sample material and (iii) using several antibodies per target protein**.** Sensitivity of the assay has been described in the lower ng/ml range by detecting prostate specific antigen (PSA) [5]. Nonetheless, sensitivity is very much dependent on the antibody (e.g. target affinity, functionality as capture reagent) as well as on the antigen (e.g. accessibility, stability, modification). For independent verification of the identified candidates, we have conducted sandwich assays using commercially available kits and therefore reagents produced outside the Human Protein Atlas. Functional sandwich assays were though not available to all targets. A next phase analysis will therefore be multiplexed sandwich immunoassays including preferentially as many of the 20 targets in one assay as possible. This is though a challenge due to detection antibody cross-reactivity [16].

Although a variety of metastases can be analyzed, our choice of primary tumors, lymph node metastases, and liver metastases was ideal to detect proteomic serum protein signatures, which were associated with early tumors and progressive stages. Moreover, our main goal was to select new potential interesting targets that could facilitate early diagnosis and monitoring of disease progression for WD-SI-NETs. Indeed, despite recent findings on putative markers [11], [17], as well as the first systematic analysis of circulating tumor cells in NETs [18], the recognition of new diagnostic biomarkers remains a challenge.

To overcome some of the major challenges associated with the identification of novel biomarkers, we combined well-defined sample collections with a highly multiplexed antibody suspension bead array to generate protein profiles in blood of WD-SI-NET patients and healthy individuals. Verification of a set of 20 protein profiles identified during the discovery phase, resulted in supportive classification performance on an independently analyzed sample cohort. In addition, the verification resulted in a more defined list of six analytes, which arose as significant classifiers of healthy controls and cancer patients, irrespectively from the disease stage. Briefly, these proteins are IGFBP2, SHKBP1, ETS1, IGF1, IL1α, and STX2. The current indications suggest that such a classification performance may provide a promising and yet relevant marker panel for future efforts towards improved detection and eventually earlier diagnosis of the disease.

Because biomarker signatures should ideally be disease-stage specific, we compared the data from individuals at different stages (PT, LNM and LM) to healthy controls, achieving more than 80% classification performance for each pairwise comparison using 20 selected antibodies. Furthermore, at the stage of primary tumors, four proteins IGF1, IL1α, SHKBP1, and EGR3 were identified as significant in the verification analysis. Similarly for patients with lymph node metastasis we identified four proteins, namely IL1α, XIAP, STX2, and SHKBP1, whereas for patients with liver metastasis we identified IGF1, IL1α, IGFBP2, MAML3, and SHKBP1 as significant.

The results of our profiling from clinical WD-SI-NETs at different stages of disease led to four potential novel marker candidates to distinguish different stages of WD-SI-NET cases from healthy subjects. Indeed, our major findings demonstrate that different targets such as IGF1, IL1α, SHKBP1, and EGR3 are pivotal to classify WD-SI-NETs at the stage of PT; IL1α, XIAP, STX2 and SHKBP1 classify LNM patients, whereas IGF1, IL1α, IGFBP2, MAML3 and SHKBP1 properly classify LM patients. We would like to describe major protein targets and extend this discussion for the remaining markers in **Text S1**.

Reassuringly, insulin-like growth factor 1 (IGF1) has been previously described as a biomarker for SI-NETs [19] indicating the validity of our approach. However, due to the paucity of information about their real function and the lack of direct correlation with WD-SI-NETs at different stages of disease these proteins require further investigation. However, these antigens highlight the importance that angiogenesis and inflammation can have in WD-SI-NETs, like they have in the course of other malignancies.

IGF1 is a protein similar to insulin in function and structure and is a member of a protein family involved in mediating growth and development [20]. The activation of the IGF1/IGF1 receptor system (IGF1/IGF1R) is a critical event in transformation and tumorigenesis in a wide variety of human tumors [21]-[23]. The IGF1/IGF1R system has been recently studied in SI-NETs [19]. Moreover, results suggest that IGF1 may play an important role even at the early stages of tumor formation [19].

Insulin-like growth factor binding protein 2 (IGFBP2) regulates the function of IGF-1. It is up-regulated in a dose-dependent manner in melanoma cells treated with IGF-1, which indicates a possible role of IGFBP2 in the pathogenesis of melanoma [5]. There is no evidence that this protein is related to the SI-NETs at the moment. However, IGFBP2 was identified in HS vs LM and showed an average AUC 0.78 in four rounds of analysis. Although this is not a perfect value, IGFBB2 maintains a significant reliability as potential diagnostic marker.

For both of the above target proteins identified differences were confirmed using sandwich immunoassays. As this dedicated assays use one antibody for capture and a second for detection, this assay is more specific then the single binder assay, which was employed during the first and highly multiplexed discovery-driven analysis. As shown also by supplementing the sandwich assays with HPA antibodies utilized during discovery, concordant trends were observed between recommended and HPA capture antibodies. Even though further optimization for IGF1 detection would be needed for a further integration of this HPA capture antibody, the results show that profiles from single binder assay can provide valuable information of differential detection and that antibodies from such screenings can be used for functional sandwich assays.

In conclusion, serum protein profiles generated by antibody suspension bead arrays identified candidate proteins assisting a classification of primary tumors, lymph-node metastases and liver metastases. The most important findings suggested that IGF1, IL1α, SHKBP1, and EGR3 were able to distinguish between controls and primary tumor-bearing patients. Further evaluation of the functional relation of the identified signatures to WD-SI-NETs using additional serum samples and tissue material, establishment of sandwich ELISAs and immunohistological assays will eventually lead to a more refined understanding of the proposed biomarker candidates for the detection and classification of WD-SI-NETs.

## Materials and Methods

### Ethics Statement

All patient and control blood samples were included in the study after a written consent statement was obtained from each individual. The study was approved by the regional ethical committee at the Clinic of Endocrine Oncology, Uppsala University Hospital, Sweden (ref. no. Dnr 2011/426).

### Samples

Serum samples were obtained at two different time points (2009 and 2012) from the Uppsala University Hospital Biobank. Samples were divided into two independent cohorts, cohort 1 and 2. Cohort 1 included 20 healthy controls (HCs), 19 untreated SI-NETs (primary tumors, PT), 19 lymph node metastases (LM) and 19 liver metastases (LNM). Cohort 2 included 36 healthy age-matched controls as well as 96 untreated WD-SI-NETs, (29 PT, 23 LNM and 44 LM). A more detailed summary of each cohort can be found in **Table 1**. All samples represent different individuals and there is no overlap between cohorts or within different patient groups.

### Antibodies

We prepared a list of potentially interesting protein targets using information from the literature and data from our published [17], [24] and unpublished WD-SI-NET microarray analyses data. Protein profiles were generated for a set of 184 antibodies targeting 124 unique proteins. A list of all unique proteins targeted can be found in **Table S3.**

### Bead coupling

Coupling of antibodies to beads was performed as previously described [25] and 30 µl of each bead identity was coupled to 1.6 µg of a different antibody. First, beads were washed twice on a magnet with activation buffer (AB) (0.1 M NaH2PO4 (Merck), pH 6.2) and subsequently beads were resuspended in 50 µl of AB. After resuspension, 50 µl of activation solution (AS) (50 mg/ml NHS (Pierce), 50 mg/ml EDC (Pierce) in AB) were added and beads were incubated for 20 min at room temperature with rotation at 650 rpm in the dark. Upon activation, beads were washed twice on a magnet with MES buffer 0.1M 2[N-Morpholino] ethanesulfonic acid (Sigma), pH 4.5 and 1.6 µg of antibody diluted in MES were added. Antibody coupling on the beads was allowed to proceed for 2 h at RT with constant rotation at 650 rpm. After coupling, beads were washed twice on a magnet with 1x PBS (Medicago) with 0.05% v/v Tween-20 (Sigma). Coupled beads are stored in 50 µl of storage buffer (Blocking reagent for ELISA (Roche) supplemented with proclin (Sigma)) at 4°C in the dark. All different bead IDs, carrying different capture antibodies were mixed to create a suspension bead array (SBA).

### Sample preparation

Prior to analysis, samples were labeled with biotin and heat-treated. Samples were centrifuged for 10 min at 3500 rpm and 7.5 µl of each sample were diluted in 55 µl of PBS. Pre-weighted NHS-biotin (2 mg, Pierce) was diluted in 200 µl of DMSO to a final concentration of 10 mg/ml. For each labeling reaction, 1.5 µl reconstituted biotin were diluted in 3.5 µl PBS and 5 µl biotin were added to 25 µl of each sample. Labeling was allowed to proceed for 2 h at 4°C and the reaction was stopped by adding 12.5 µl 0.5 M Tris-HCl. Labeled samples were diluted 1∶50 in assay buffer (0.5% (w/v) polyvinyl alcohol and 0.8% (w/v) polyvinylpyrrolidone (Sigma) in 0.1% casein in PBS supplemented with 0.5 mg/ml rabbit IgG (Bethyl Laboratories) and heat-treated for 30 min at 56°C.

### Sample analysis

Next 45 µl of each sample were mixed with 5 µl SBA and allowed to incubate overnight at RT with constant rotation at 650 rpm. After incubation, beads were washed three times on a magnet with PBS-T and 50 µl 0.4% PFA were added in each well. Beads in PFA were incubated for 10 min at RT with constant rotation prior to being washed once with PBS-T. Streptavidin R-PE (SAPE, Invitrogen) was diluted 1∶600 in PBS and 50 µl were added in each well. SAPE binding to biotinylated captured protein molecules was allowed to proceed for 20 min at RT with constant rotation. Prior to analysis in a Luminex FlexMap3D instrument, beads were washed three times on a magnet with PBS-T. Median fluorescence intensities (MFI) of each bead ID were used for subsequent analysis.

### Sandwich immunoassay

A pair of antibodies for sandwich immunoassay analysis of IGFBP2 and IGF1 was acquired (RnD Systems). For capture 500,000 beads (MagPlex, Luminex) were coupled either with 4 µg of monoclonal antibody (RnD Systems) or 1.6 µg of HPA antibodies using the same procedure as described above and coupling was confirmed by with R-phycoerythrin labeled anti-mouse antibody coupled or R-phycoerythrin labeled anti-rabbit antibody (both Moss Inc). For IGFBP2, samples were diluted 1∶10 in 5% Tween20 in PBS and for IGF1 1∶2 in a buffer containing 0.5% (w/v) polyvinyl alcohol and 0.8% (w/v) polyvinylpyrrolidone in 0.1% casein in PBS (all Sigma). Both assays were conducted as 2-plex assay using 5 µl beads and 45 µl diluted serum sample. The assays were incubated 3 h at RT for IGFBP2 and overnight for IGF1, beads were washed 3x 100 µl of PBS-T and on a magnet, followed by adding 25 µl biotinylated detection antibody with IGFBP2 at 0.2 µg/ml and IGF1 at 0.5 µg/ml. Both detection antibodies were incubated for 1 h at RT and after 3x 100 µl PBS-T washing 50 µl Streptavidin R-PE (SAPE, Invitrogen) diluted at 1∶750 in PBS-Twas added and incubated 30 min. Beads were washed again and measured in 100 µl PBS-T using the Luminex FlexMap3D instrument.

### Data Analysis

All data analysis was performed using the R statistical software [26]. MFI for each bead ID and for each sample were initially normalized using probabilistic quotient normalization (PQN) [27], [28].

Prior to multivariate analysis with random forest (RF) [7] or BGA [8] normalized intensities were scaled and centered to account for differences of absolute intensity values between different experiments. RF analysis was performed using the randomForest package [7] and proximity matrixes generated by RF were further scaled in two dimensions and plotted using the MDSplot function in R. Nearest neighbor assignment of sample identities was performed using a majority vote among five neighbors (closest data point in Euclidean distance) for each sample, using the package class [29], [30]. Each RF analysis was performed 1000 times, using default parameters and the classification accuracy reported correspond to the median classification accuracy of the 1000 repeated classifications.

Individual significant analytes were identified using a Wilcoxon rank-sum test with a cut-off for significance set at 0.01, without multiple sample testing correction. AUC values were calculated using the package pROC [31].

For sandwich immunoassay, a linear model was used on the randomized samples to account for intensity differences due to the sequence of measurement (location of sample in plate).

## Supporting Information

Text S1 (DOCX)

Table S1 Raw data of antibody suspension bead array.
(XLS)

Table S2 List of all 20 analytes selected during the discovery phase.
(DOCX)

Table S3 List of all analytes targeted in the discovery phase.
(DOCX)

## References

[1] Kloppel G, Couvelard A, Perren A, Komminoth P, McNicol AM, et al (2009) ENETS Consensus Guidelines for the Standards of Care in Neuroendocrine Tumors: towards a standardized approach to the diagnosis of gastroenteropancreatic neuroendocrine tumors and their prognostic stratification. Neuroendocrinology 90: 162–166. 19060454 10.1159/000182196

[2] Modlin IM, Moss SF, Chung DC, Jensen RT, Snyderwine E (2008) Priorities for improving the management of gastroenteropancreatic neuroendocrine tumors. J Natl Cancer Inst 100: 1282–1289. 18780869 10.1093/jnci/djn275 PMC2538549

[3] Neiman M, Hedberg JJ, Donnes PR, Schuppe-Koistinen I, Hanschke S, et al (2011) Plasma profiling reveals human fibulin-1 as candidate marker for renal impairment. Journal of proteome research 10: 4925–4934. 21888404 10.1021/pr200286c

[4] Schwenk JM, Gry M, Rimini R, Uhlen M, Nilsson P (2008) Antibody suspension bead arrays within serum proteomics. Journal of proteome research 7: 3168–3179. 18588325 10.1021/pr700890b

[5] Schwenk JM, Igel U, Neiman M, Langen H, Becker C, et al (2010) Toward next generation plasma profiling via heat-induced epitope retrieval and array-based assays. Molecular & cellular proteomics : MCP 9: 2497–2507. 20682762 10.1074/mcp.M110.001560 PMC2984230

[6] Uhlen M, Ponten F (2005) Antibody-based proteomics for human tissue profiling. Molecular & cellular proteomics : MCP 4: 384–393. 15695805 10.1074/mcp.R500009-MCP200

[7] Breiman L (2001) Random Forests. Machine Learning 45: 5–32.

[8] Culhane AC, Perriere G, Considine EC, Cotter TG, Higgins DG (2002) Between-group analysis of microarray data. Bioinformatics 18: 1600–1608. 12490444 10.1093/bioinformatics/18.12.1600

[9] Lindholm DP, Oberg K (2011) Biomarkers and molecular imaging in gastroenteropancreatic neuroendocrine tumors. Horm Metab Res 43: 832–837. 22009449 10.1055/s-0031-1287794

[10] Oberg K (2011) Circulating biomarkers in gastroenteropancreatic neuroendocrine tumours. Endocr Relat Cancer 18 Suppl 1S17–25. 22005113 10.1530/ERC-10-0280

[11] Cui T, Hurtig M, Elgue G, Li SC, Veronesi G, et al (2010) Paraneoplastic antigen Ma2 autoantibodies as specific blood biomarkers for detection of early recurrence of small intestine neuroendocrine tumors. PLoS One 5: e16010. 21209860 10.1371/journal.pone.0016010 PMC3012732

[12] Cui T, Tsolakis AV, Li SC, Cunningham JL, Lind T, et al.. (2012) Olfactory Receptor 51E1 Protein as a Potential Novel Tissue Biomarker for Small Intestine Neuroendocrine Carcinomas. Eur J Endocrinol. 10.1530/EJE-12-0814 23184910

[13] Li SC, Essaghir A, Martijn C, Lloyd RV, Demoulin JB, et al.. (2013) Global microRNA profiling of well-differentiated small intestinal neuroendocrine tumors. Mod Pathol. 10.1038/modpathol.2012.216 PMC3647117 23328977

[14] Sjoberg R, Sundberg M, Gundberg A, Sivertsson A, Schwenk JM, et al.. (2011) Validation of affinity reagents using antigen microarrays. New biotechnology. 10.1016/j.nbt.2011.11.009 22134247

[15] Asplund A, Edqvist PH, Schwenk JM, Ponten F (2012) Antibodies for profiling the human proteome-The Human Protein Atlas as a resource for cancer research. Proteomics 12: 2067–2077. 22623277 10.1002/pmic.201100504

[16] Poetz O, Henzler T, Hartmann M, Kazmaier C, Templin MF, et al (2010) Sequential multiplex analyte capturing for phosphoprotein profiling. Molecular & cellular proteomics : MCP 9: 2474–2481. 20682761 10.1074/mcp.M110.002709 PMC2984240

[17] Li SC, Martijn C, Cui T, Essaghir A, Luque RM, et al (2012) The somatostatin analogue octreotide inhibits growth of small intestine neuroendocrine tumour cells. PloS one 7: e48411. 23119007 10.1371/journal.pone.0048411 PMC3485222

[18] Khan MS, Tsigani T, Rashid M, Rabouhans JS, Yu D, et al (2011) Circulating tumor cells and EpCAM expression in neuroendocrine tumors. Clin Cancer Res 17: 337–345. 21224371 10.1158/1078-0432.CCR-10-1776

[19] Kaltsas GA, Cunningham JL, Falkmer SE, Grimelius L, Tsolakis AV (2011) Expression of connective tissue growth factor and IGF1 in normal and neoplastic gastrointestinal neuroendocrine cells and their clinico-pathological significance. Endocr Relat Cancer 18: 61–71. 20959439 10.1677/ERC-10-0026

[20] Mole D, Gagliano T, Gentilin E, Tagliati F, Pasquali C, et al (2011) Targeting protein kinase C by Enzastaurin restrains proliferation and secretion in human pancreatic endocrine tumors. Endocr Relat Cancer 18: 439–450. 21606156 10.1530/ERC-11-0055

[21] Ewing GP, Goff LW (2010) The insulin-like growth factor signaling pathway as a target for treatment of colorectal carcinoma. Clin Colorectal Cancer 9: 219–223. 20920993 10.3816/CCC.2010.n.032

[22] Tsugane S, Inoue M (2010) Insulin resistance and cancer: epidemiological evidence. Cancer Sci 101: 1073–1079. 20345478 10.1111/j.1349-7006.2010.01521.x PMC11159937

[23] Hilmi C, Larribere L, Giuliano S, Bille K, Ortonne JP, et al (2008) IGF1 promotes resistance to apoptosis in melanoma cells through an increased expression of BCL2, BCL-X(L), and survivin. J Invest Dermatol 128: 1499–1505. 18079751 10.1038/sj.jid.5701185

[24] Leja J, Essaghir A, Essand M, Wester K, Oberg K, et al (2009) Novel markers for enterochromaffin cells and gastrointestinal neuroendocrine carcinomas. Mod Pathol 22: 261–272. 18953328 10.1038/modpathol.2008.174

[25] Haggmark A, Neiman M, Drobin K, Zwahlen M, Uhlen M, et al (2012) Classification of protein profiles from antibody microarrays using heat and detergent treatment. New biotechnology 29: 564–570. 22023822 10.1016/j.nbt.2011.10.005

[26] Ihaka R, Gentleman R (1996) R: a language for data analysis and graphics. Journal of Computional and Graphical Statistics 5: 299–3214.

[27] Dieterle F, Ross A, Schlotterbeck G, Senn H (2006) Probabilistic quotient normalization as robust method to account for dilution of complex biological mixtures. Application in ^1^H NMR metabonomics. Analytical Chemistry 78: 4281–4290. 16808434 10.1021/ac051632c

[28] Kato BS, Nicholson G, Neiman M, Rantalainen M, Holmes CC, et al (2011) Variance decomposition of protein profiles from antibody arrays using a longitudinal twin model. Proteome science 9: 73. 22093360 10.1186/1477-5956-9-73 PMC3247853

[29] Ripley BD (1996) Pattern recognition and neural networks. Cambridge ; New York: Cambridge University Press. xi, 403 p.p.

[30] Venables WN, Ripley BD (1999) Modern applied statistics with S-PLUS. New York: Springer. xi, 501 p. p.

[31] Robin X, Turck N, Hainard A, Tiberti N, Lisacek F, et al (2011) pROC: an open-source package for R and S+ to analyze and compare ROC curves. BMC Bioinformatics 12: 77. 21414208 10.1186/1471-2105-12-77 PMC3068975

