# Supplementary material for: Identification of Candidate Serum Proteins for Classifying Well-Differentiated Small Intestinal Neuroendocrine Tumors
Source: PLoS One. 2013 Nov 25;8(11):e81712. doi: 10.1371/journal.pone.0081712 (PMC3839889; doi:10.1371/journal.pone.0081712)
Supplement: Text S1 — (DOCX) [file pone.0081712.s001.docx]

**Supporting Information**

In addition to the main targets, we further discuss the remaining protein of interest here.

First, the early growth response protein 3 (EGR3) encodes a transcriptional regulator belonging to the EGR family of C2H2-type zinc-finger proteins, an early growth response gene, which may be induced by mitogenic stimulation. In addition, it may also play a role in a wide variety of processes including endothelial cell growth and migration, and neuronal development. The inducible zinc-finger transcription factors EGR1, EGR2, and EGR3 regulate the expression of numerous genes involved in differentiation, growth, and response to extracellular signals [[1](#_ENREF_1)]. Far less has been elucidated regarding the role of this protein to endocrine cells. Lately, it has been established that miR-210 is induced by hypoxia and plays different roles in the development of certain cancers. In addition, evidence suggested that miR-210 expression in pancreatic cancers is induced by hypoxia through an HIF-1alpha-dependent pathway, without influencing pancreatic cancer cell proliferation. Moreover, many genes including EGR3 may be potential miR-210 targets in pancreatic cancer cells [[2](#_ENREF_2)].

Second, the official full name of XIAP is X-linked inhibitor of apoptosis. This gene encodes a protein that belongs to a family of apoptotic suppressor proteins. Members of this family share a conserved motif termed baculovirus IAP repeat which is necessary for their anti-apoptotic function. Although there are not direct links to gastrointestinal neuroendocrine tumors, Hiscutt et al. reported on the prognostic significance of the inhibitor of XIAP protein in melanoma [[3](#_ENREF_3)]. It should be noted that although XIAP is a unique marker for the classification of LNM patients, this marker is not a good classifier when used by itself with an average AUC for all different runs of cohort 1 and 2 of 0.6.

Third, syntaxin2 (STX2) belongs to a large protein family, named syntaxin/epimorphin protein, which is implicated in the targeting and fusion of intracellular transport vesicles. The product of this gene regulates epithelial-mesenchymal interactions and epithelial cell morphogenesis and activation. Although the function of syntaxin 2 remains unclear, mutants may block synaptic release in neurons and norepinephrine release in neuroendocrine cells and this suggests a potential pivotal role of STX2 for Ca2^+^-triggered exocytosis. Indeed, mutants of the functional protein mechanism can generate syntaxin-free cells and may lead to pharmaceuticals that target syntaxin selectively [[4](#_ENREF_4)].

Interleukin-1 alpha (IL1a) is a cytokine with pleiotropic functions in immune response, inflammation, and hematopoiesis. Several publications have provided insight into the biology of IL-1 molecules [[5-7](#_ENREF_5)], which has never been correlated to either endothelial cells or NET cells. However, it has been shown that the upregulation of endothelial adhesion molecules is a sign of endothelial cell activation in inflammatory responses. These findings may support the idea that tumor angiogenesis induces endothelial cells energy, acting as a tumor-protecting mechanism [[8](#_ENREF_8)].

SH3KBP1-binding protein 1 (SHKBP1) is a recently identified protein, which prevents epidermal growth factor receptor (EGFR) degradation by disrupting the 85-kDa Cbl-interacting protein (CIN85) complex (c-Cbl-CIN85) [[9](#_ENREF_9)]. The binding of CIN85 to c-Cbl is critical for endocytosis and degradation of EGFR [[10](#_ENREF_10)]. Although little is currently known about protein function in most neuroendocrine tumors, SHKBP1 could also promote the EGFR signaling pathway in SI-NET by interrupting c-Cbl-CIN85 complex and inhibiting EGFR degradation [[9](#_ENREF_9)].

Mastermind-like protein 3 (MAML3), which belongs to the Mastermind-like (MAML) protein family, is a pivotal transcription factor in the control of Notch signaling, a relevant pathway correlated to solid tumor progression. The interaction among multiple signaling pathways, including Wnt/Wg, Shh/Hh, BMP, and Notch also orchestrate mitosis, motility, and differentiation of the human intestine [[11](#_ENREF_11)]. Thus, Notch activation relates to tumor progressing functions, which have been detected in two aggressive types of human cancer, such as pancreatic adenocarcinoma and basal type of breast [[12](#_ENREF_12)]. Moreover, novel studies reported the importance of MAML3 in controlling acetylation and de-acetylation of different proteins via SUMOtylation [[13](#_ENREF_13)], which influences histone deacetylase 7 (HDAC7) [[14](#_ENREF_14)].

1. Suehiro J, Hamakubo T, Kodama T, Aird WC, Minami T (2010) Vascular endothelial growth factor activation of endothelial cells is mediated by early growth response-3. Blood 115: 2520-2532.

2. Chen WY, Liu WJ, Zhao YP, Zhou L, Zhang TP, et al. (2012) Induction, modulation and potential targets of miR-210 in pancreatic cancer cells. Hepatobiliary Pancreat Dis Int 11: 319-324.

3. Hiscutt EL, Hill DS, Martin S, Kerr R, Harbottle A, et al. (2010) Targeting X-linked inhibitor of apoptosis protein to increase the efficacy of endoplasmic reticulum stress-induced apoptosis for melanoma therapy. J Invest Dermatol 130: 2250-2258.

4. Wang D, Zhang Z, Dong M, Sun S, Chapman ER, et al. (2011) Syntaxin requirement for Ca2+-triggered exocytosis in neurons and endocrine cells demonstrated with an engineered neurotoxin. Biochemistry 50: 2711-2713.

5. Rider P, Carmi Y, Guttman O, Braiman A, Cohen I, et al. (2011) IL-1alpha and IL-1beta recruit different myeloid cells and promote different stages of sterile inflammation. J Immunol 187: 4835-4843.

6. Enya K, Hayashi H, Takii T, Ohoka N, Kanata S, et al. (2008) The interaction with Sp1 and reduction in the activity of histone deacetylase 1 are critical for the constitutive gene expression of IL-1 alpha in human melanoma cells. J Leukoc Biol 83: 190-199.

7. Kholmanskikh O, van Baren N, Brasseur F, Ottaviani S, Vanacker J, et al. (2010) Interleukins 1alpha and 1beta secreted by some melanoma cell lines strongly reduce expression of MITF-M and melanocyte differentiation antigens. Int J Cancer 127: 1625-1636.

8. Griffioen AW, Damen CA, Blijham GH, Groenewegen G (1996) Tumor angiogenesis is accompanied by a decreased inflammatory response of tumor-associated endothelium. Blood 88: 667-673.

9. Feng L, Wang JT, Jin H, Qian K, Geng JG (2011) SH3KBP1-binding protein 1 prevents epidermal growth factor receptor degradation by the interruption of c-Cbl-CIN85 complex. Cell Biochem Funct 29: 589-596.

10. Kowanetz K, Szymkiewicz I, Haglund K, Kowanetz M, Husnjak K, et al. (2003) Identification of a novel proline-arginine motif involved in CIN85-dependent clustering of Cbl and down-regulation of epidermal growth factor receptors. J Biol Chem 278: 39735-39746.

11. Takashima S, Hartenstein V (2012) Genetic control of intestinal stem cell specification and development: a comparative view. Stem Cell Rev 8: 597-608.

12. Brabletz S, Bajdak K, Meidhof S, Burk U, Niedermann G, et al. (2011) The ZEB1/miR-200 feedback loop controls Notch signalling in cancer cells. EMBO J 30: 770-782.

13. Lindberg MJ, Popko-Scibor AE, Hansson ML, Wallberg AE (2010) SUMO modification regulates the transcriptional activity of MAML1. FASEB J 24: 2396-2404.

14. Barneda-Zahonero B, Parra M (2012) Histone deacetylases and cancer. Mol Oncol.
