# Supplementary material for: Identification of Candidate Serum Proteins for Classifying Well-Differentiated Small Intestinal Neuroendocrine Tumors
Source: PLoS One. 2013 Nov 25;8(11):e81712. doi: 10.1371/journal.pone.0081712 (PMC3839889; doi:10.1371/journal.pone.0081712)
Supplement: Table S2 — List of all 20 analytes selected during the discovery phase. (DOCX) [file pone.0081712.s003.docx]

**Supporting Table S2.** List of all 20 analytes selected during the discovery phase.

| **#** | **Bead ID** | **Antibody ID** | **Gene name** | **Ensembl** | **UniProt** |
| --- | --- | --- | --- | --- | --- |
| 1 | 1 | HPA041522 | NOB1 | ENSG00000141101 | Q9ULX3 |
| 2 | 3 | HPA041887 | SHKBP1 | ENSG00000160410 | Q8TBC3 |
| 3 | 17 | HPA042428 | XIAP | ENSG00000101966 | P98170 |
| 4 | 106 | HPA045140 | IGFBP2 | ENSG00000115457 | P18065 |
| 5 | 118 | HPA047675 | GNA12 | ENSG00000146535 | Q03113 |
| 6 | 120 | HPA048124 | AC008537.4, CYP2B6 | ENSG00000256612, ENSG00000197408 | P20813 |
| 7 | 122 | HPA048546 | IL1A | ENSG00000115008 | P01583 |
| 8 | 123 | HPA048549 | TSC1 | ENSG00000165699 | Q92574 |
| 9 | 129 | HPA048946 | IGF1 | ENSG00000017427 | P05019 |
| 10 | 186 | HPA006290 | PCDH17 | ENSG00000118946 | O14917 |
| 11 | 187 | HPA006206 | EGR3 | ENSG00000179388 | Q06889 |
| 12 | 191 | HPA007616 | LCT | ENSG00000115850 | P09848 |
| 13 | 208 | HPA015037 | ARID5B | ENSG00000150347 | Q14865 |
| 14 | 209 | HPA015703 | CRH | ENSG00000147571 | P06850 |
| 15 | 230 | HPA019786 | ETS1 | ENSG00000134954 | P14921 |
| 16 | 260 | HPA029731 | GATA3 | ENSG00000107485 | P23771 |
| 17 | 266 | HPA028904 | VEGFC | ENSG00000150630 | P49767 |
| 18 | 293 | HPA037718 | MAML3 | ENSG00000196782 | Q96JK9 |
| 19 | 297 | HPA038675 | RHOF | ENSG00000139725 | Q9HBH0 |
| 20 | 304 | HPA038189 | STX2 | ENSG00000111450 | P32856 |
