# Supplementary material for: Identification of Candidate Serum Proteins for Classifying Well-Differentiated Small Intestinal Neuroendocrine Tumors
Source: PLoS One. 2013 Nov 25;8(11):e81712. doi: 10.1371/journal.pone.0081712 (PMC3839889; doi:10.1371/journal.pone.0081712)
Supplement: Table S3 — List of all analytes targeted in the discovery phase. (DOCX) [file pone.0081712.s004.docx]

**Supporting Table S3.** List of all analytes targeted in the discovery phase.

| **#** | **Gene** | **Uniprot assecion number** |
| --- | --- | --- |
| 1 | ABCG5 | Q9H222 |
| 2 | AC008537.2, CYP2B6 | P20813 |
| 3 | ALPP, ALPPL2, ALPI | P05187, P10696, P09923 |
| 4 | ANGPT2 | O15123 |
| 5 | AQP12B, AQP12A | A6NM10, Q8IXF9 |
| 6 | ARID5B | Q14865 |
| 7 | ARL14 | Q8N4G2 |
| 8 | ATP6V1E1 | P36543 |
| 9 | ATP8B1 | O43520 |
| 10 | BCL2L1 | Q07817 |
| 11 | BIRC5 | O15392 |
| 12 | BIRC8, XIAP | Q96P09, P98170 |
| 13 | BRCA1 | P38398 |
| 14 | CCND1 | P24385 |
| 15 | CCNE1 | P24864 |
| 16 | CDKN1B | P46527 |
| 17 | CELSR3 | Q9NYQ7 |
| 18 | CHGA | P10645 |
| 19 | CHGB | P05060 |
| 20 | COBL | O75128 |
| 21 | CREB3L3 | Q68CJ9 |
| 22 | CRH | P06850 |
| 23 | CRP | P02741 |
| 24 | CSNK1G1 | Q9HCP0 |
| 25 | CUEDC1 | Q9NWM3 |
| 26 | CXCR4 | P61073 |
| 27 | CYB5A | P00167 |
| 28 | CYTH2 | Q99418 |
| 29 | DDX21 | Q9NR30 |
| 30 | DDX56 | Q9NY93 |
| 31 | DHPS | P49366 |
| 32 | DHX33 | Q9H6R0 |
| 33 | DRD5 | P21918 |
| 34 | DSP | P15924 |
| 35 | EEF2 | P13639 |
| 36 | EGR3 | Q06889 |
| 37 | ETFDH | Q16134 |
| 38 | **ETS1** | P14921 |
| 39 | FAM155B | O75949 |
| 40 | FEN1 | P39748 |
| 41 | FGD4 | Q96M96 |
| 42 | FNIP2 | Q9P278 |
| 43 | FOXP4 | Q8IVH2 |
| 44 | GATA3 | P23771 |
| 45 | GGT7 | Q9UJ14 |
| 46 | GHSR | Q92847 |
| 47 | GNA12 | Q03113 |
| 48 | GPR112 | Q8IZF6 |
| 49 | GRIA2 | P42262 |
| 50 | HDAC8 | Q9BY41 |
| 51 | HN1L | Q9H910 |
| 52 | HSF5 | Q4G112 |
| 53 | IGF1 | P05019 |
| 54 | IGF1R | P08069 |
| 55 | **IGFBP2** | P18065 |
| 56 | IGFBP5 | P24593 |
| 57 | IL1A | P01583 |
| 58 | ITGA6 | P23229 |
| 59 | JOSD1 | Q15040 |
| 60 | KISS1R | Q969F8 |
| 61 | KLB | Q86Z14 |
| 62 | LCT | P09848 |
| 63 | LGR4 | Q9BXB1 |
| 64 | MAML3 | Q96JK9 |
| 65 | MATN2 | O00339 |
| 66 | MED12L | Q86YW9 |
| 67 | MKL2 | Q9ULH7 |
| 68 | MLLT11 | Q13015 |
| 69 | MOB1A, MOB1B | Q9H8S9, Q7L9L4 |
| 70 | NAF1 | Q96HR8 |
| 71 | NDC80 | O14777 |
| 72 | NKX2-2 | O95096 |
| 73 | NKX2-3 | Q8TAU0 |
| 74 | NOB1 | Q9ULX3 |
| 75 | NOTCH2 | Q04721 |
| 76 | NOTCH2, NOTCH2NL, RP11-458D21.5 | Q04721, Q7Z3S9 |
| 77 | NOTCH3 | Q9UM47 |
| 78 | NPC1L1 | Q9UHC9 |
| 79 | NUPL1 | Q9BVL2 |
| 80 | OTC | P00480 |
| 81 | PCDH17 | O14917 |
| 82 | PDILT | Q8N807 |
| 83 | PLEKHA7 | Q6IQ23 |
| 84 | PLS1, LCP1, PLS3 | Q14651, P13796, P13797 |
| 85 | PLXND1 | Q9Y4D7 |
| 86 | PNMA2 | Q9UL42 |
| 87 | PRKCA | P17252 |
| 88 | PRKD2 | Q9BZL6 |
| 89 | RAB28 | P51157 |
| 90 | RANBP1 | P43487 |
| 91 | RHOF | Q9HBH0 |
| 92 | RIMKLA | Q8IXN7 |
| 93 | RIMS2 | Q9UQ26 |
| 94 | ROR2 | Q01974 |
| 95 | RPSA, RPSAP58 | P08865 |
| 96 | SGCE | O43556 |
| 97 | SGK1 | O00141 |
| 98 | SHKBP1 | Q8TBC3 |
| 99 | SLC10A2 | Q12908 |
| 100 | SLC13A1 | Q9BZW2 |
| 101 | SLC6A19 | Q695T7 |
| 102 | SMPD3 | Q9NY59 |
| 103 | SMPDL3A | Q92484 |
| 104 | SPATA6 | Q9NWH7 |
| 105 | STX2 | P32856 |
| 106 | SYNJ2BP | P57105 |
| 107 | TBC1D19 | Q8N5T2 |
| 108 | TCF25 | Q9BQ70 |
| 109 | THNSL2 | Q86YJ6 |
| 110 | TLE2 | Q04725 |
| 111 | TMEM199 | Q8N511 |
| 112 | TOX | O94900 |
| 113 | TPH1 | P17752 |
| 114 | TPST1 | O60507 |
| 115 | TRIM13 | O60858 |
| 116 | TSC1 | Q92574 |
| 117 | TSC2 | P49815 |
| 118 | TUBAL3 | A6NHL2 |
| 119 | UGT2B17, UGT2B15, UGT2B10, UGT2A3, UGT2B7, UGT2B11, UGT2B28, UGT2B4, UGT2A1 | O75795, P54855, P36537, Q6UWM9, P16662, O75310, Q9BY64, P06133, Q9Y4X1 |
| 120 | VEGFC | P49767 |
| 121 | VEZT | Q9HBM0 |
| 122 | WRB | O00258 |
| 123 | XIAP | P98170 |
| 124 | ZEB1 | P37275 |
